# A novel rapid measurement method of cervical sagittal parameters based on the integrated inclinometer of a smartphone: a validity and reliability study

**DOI:** 10.1080/07853890.2023.2289590

**Published:** 2023-12-08

**Authors:** Tianji Huang, Chunyang Zhang, Zhenghan Han, Weiyang Zhong, Zenghui Zhao, Yong Zhu, Xiaoji Luo, Jie Zhang

**Affiliations:** a Department of Orthopedic Surgery, The First Affiliated Hospital of Chongqing Medical University, Chongqing, People’s Republic of China; b Orthopedic Laboratory of Chongqing Medical University, Chongqing, People’s Republic of China; c Department of Orthopedic Surgery, People’s Hospital of Chongqing Banan District, Chongqing, People’s Republic of China; d Department of Oncology, The First Affiliated Hospital of Chongqing Medical University, Chongqing, People’s Republic of China

**Keywords:** Smartphone, measurement, cobb angle, T1S, NT

## Abstract

**Objectives:** A new method was introduced using a smartphone’s integrated inclinometer for rapid measurement of sagittal cervical parameters. The present study aims to compare the validity and reliability of the proposed method.

**Methods:** We retrospectively reviewed 120 patients with cervical spondylosis treated at our hospital. The C0-2 Cobb angle, C2-7 Cobb angle, T1-slope (T1S), and neck tilt (NT) were selected as representative sagittal angles for this study. Two methods, the smartphone’s integrated inclinometer and picture archiving and communication system (PACS), were used to measure these four representative angles. Validity, reliability, and measurement times were recorded and compared.

**Results:** The representative parameters (C0-2 Cobb angle, C2-7 Cobb angle, T1S, and NT), the ICC was 0.957 (0.939–0.970), 0.971 (0.958–0.979), 0.974 (0.963–0.982) and 0.949 (0.927–0.964) for validity respectively. For the aforementioned representative parameters, the ICC values were 0.972 (0.960–0.980), 0.979 (0.969–0.985), 0.972 (0.959–0.980), 0.942 (0.917–0.959) for intraobserver reliability respectively. For the representative parameters mentioned above, the ICC values were 0.947 (0.926–0.963), 0.964 (0.949–0.975), 0.956 (0.938-0.969), 0.916 (0.881–0.940) for interobserver reliability respectively. For the validity of the representative parameters mentioned above, the Bland-Altman plot displayed a mean difference of 0.2, 0.1, 0.1, and 0.4°with a 95% CI of 4.3, 4.5, 3.4, and 4.1°, respectively. The measurement by smartphone’s integrated inclinometer (46.31 ± 3.99 s) was significantly quicker than that by PACS (69.48 ± 3.25 s) according to independent-samples T test (*p* < 0.001).

**Conclusion:** This novel smartphone measurement based on the integrated inclinometer is accurate and reliable for measuring cervical sagittal parameters rapidly and conveniently.

## KEY MESSAGES

Sagittal parameters of the cervical spine have been widely applied in many types of spinal disorders for disease diagnosis, assessment, classification, treatment choice, and follow-up, so accurate, rapid, and convenient measurement of cervical sagittal parameters is important.

Traditional measurement of sagittal parameters of the cervical spine have their own disadvantages.

This novel smartphone measurement based on the integrated inclinometer is accurate and reliable for measuring cervical sagittal parameters rapidly and conveniently.

## 1. Introduction

Sagittal parameters of the cervical spine have been widely applied in many types of spinal disorders for disease diagnosis, assessment, classification, treatment choice, and follow-up [1–4]. One study revealed that in asymptomatic volunteers, the C0-2 Cobb angle decreased gradually with increasing age while the C2-7 cobb angle, T1-slope (T1S), and neck tilt (NT) increased gradually with increasing age, indicating that the closer the postoperative C2-7 cobb angle was to the theoretical value of the corresponding age, the better the outcomes of subaxial cervical spine surgery [5]. Wang et al. used T1S, C2-7 Cobb angle, and C0-2 Cobb angle to evaluate the outcomes of the expansiveness of open-door laminoplasty for the management of upper cervical spinal stenosis [6]. One study suggested that cervical sagittal parameters have a vital effect on the occurrence of axial neck pain in patients with cervical kyphosis, and a greater T1S may result in the development of neck pain [7]. Lee et al. suggested that NT had a significant effect when maintaining balance of the head and cervical spine, based on the finding that NT remained stable even when kyphosis was corrected by surgery [8].

Therefore, accurate, rapid, and convenient measurement of cervical sagittal parameters is important. Traditional measurement using a protractor and marker pen and the picture archiving and communication system (PACS) method (regarded as the golden standard) have their own disadvantages, while weaknesses have also been revealed for smartphone measurement methods with specific apps invented in recent years [9–10]. The inclinometer can be used to measure the inclination of a smartphone and is rooted in almost every smartphone as an intrinsic function [11]. A new method was introduced using the smartphone’s integrated inclinometer for the rapid measurement of sagittal cervical parameters. The present study aims to compare the validity and reliability of the proposed method. The measurement times using both methods were also recorded and compared.

## 2. Materials and methods

This study conformed to the relevant regulations and guidelines. The Institutional Review Board of The First Affiliated Hospital of Chongqing Medical University approved this study and waived the requirement for informed consent.

### 2.1. Patients enrollment

We chose the true lateral X-ray films, whose upper and lower edges of the vertebra are shown as one line. Lateral plain films from 120 patients with cervical spondylosis (myelopathy or radiculopathy) admitted to our hospital were retrospectively reviewed. Lateral X-rays with adequate clarity to determine the measurement points were the only inclusion criteria. The exclusion criterion was cervical kyphotic deformities.

### 2.2. Measure methods

**Figure 1.**
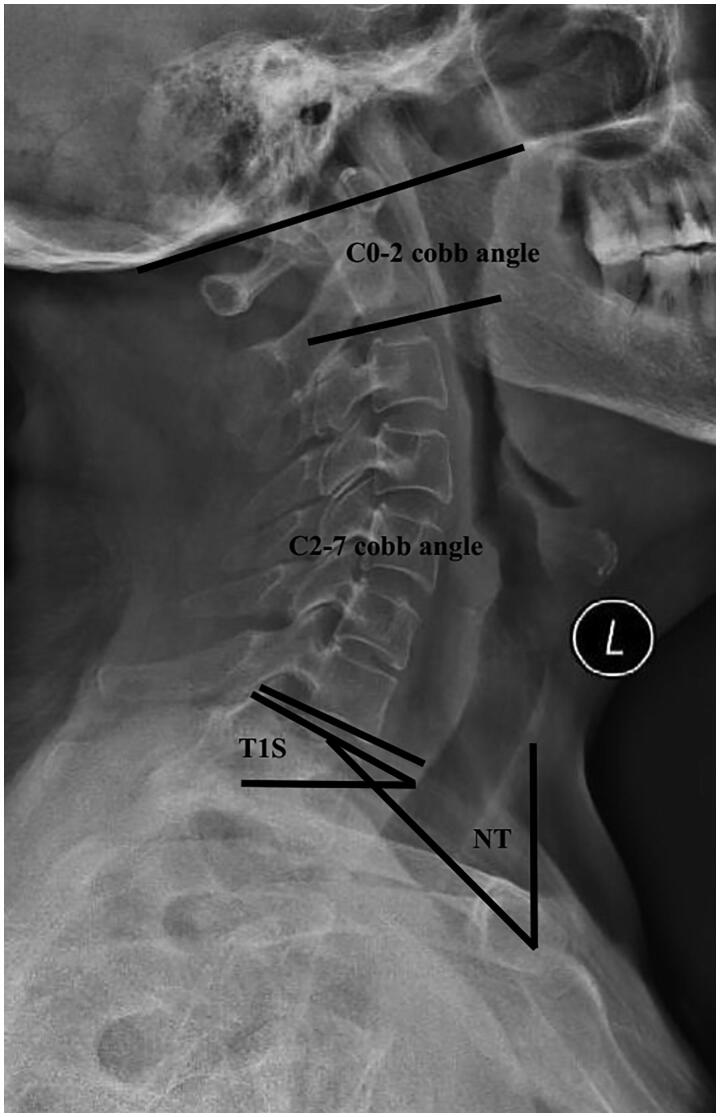
The measurement of the represented angles.

The C0-2 Cobb angle, C2-7 cobb angle, T1S, and NT were selected as representative sagittal angles for this study. The definitions of these four angles were based on previous literature and our previous study [6,12–15] (Figure 1).

**Figure 2.**
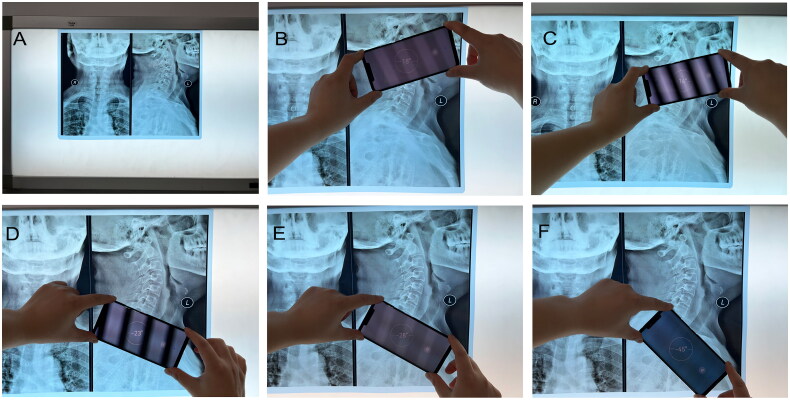
Measurement of the represented angles by smartphone’s intrinsic inclinometer. A. X-rays were placed on the film viewer, which was placed on the wall. B. Confirm the McGregor line, align a certain edge of the smartphone to the McGregor line, and record the angle displayed on the smartphone screen. C. Confirm the lower endplate of C2, align the same edge of the smartphone to the lower endplate of C2, and record the angle displayed on the smartphone screen. D. Confirm the lower endplate of C7, align the same edge of the smartphone to the lower endplate of C7, and record the angle displayed on the smartphone screen. E. Confirm the upper endplate of T1, align the same edge of the smartphone to the upper endplate of T1, and record the angle displayed on the smartphone screen. F. The line connecting the upper end of the sternum and the center of the T1 upper endplate aligned the same edge of the smartphone to this line and recorded the angle shown on the smartphone screen.

We used the same smartphone and the same computer to minimize potential errors in the presented research. The smartphone used in the study is a HUAWEI Mate 20 Pro. The resolutions of the three Leica rear cameras are 40 million, 20 million and 8 million pixels. The built-in PACS system was utilized for the measurement of the four representative angles by marking lines according to the aforementioned points, and the system can automatically display the size of the angles. An intrinsic inclinometer was used to measure these four angles on the radiographic films. The X-ray was placed on the film viewer, which was placed on the wall to ensure that the X-ray was plumbed [12] (Figure 2A).

The measurement method was similar to our previous study [11–12].

To measure the C0-2 Cobb angle, first, the McGregor line was confirmed, one edge of the smartphone was aligned to the McGregor line, and the angle shown on the smartphone screen was recorded; second, the lower endplate of C2 was confirmed, the same edge of the smartphone was aligned to the lower endplate of C2, and the angle shown on the smartphone screen was recorded, and the difference between the two angles was calculated, and then recorded as the C0-2 Cobb angle [11–12] (Figure 2B, C).

To measure C2-7 cobb angle, we first confirmed the lower endplate of C2, one edge of the smartphone was aligned to the lower endplate of C2, and recorded the angle shown on the smartphone screen; second, we confirmed the lower endplate of C7, the same edge of the smartphone was aligned to the lower endplate of C7, and recorded the angle shown on the smartphone screen; finally, the difference between the two angles was calculated, and then recorded as the C2-7 Cobb angle [11–12] (Figure 2C, D).

To measure T1S, the upper endplate of T1 was confirmed, one edge of the smartphone was aligned to the upper endplate of T1, and the absolute value of this angle displayed on the smartphone screen was recorded as T1S (because the horizontal line is always represented as 0°) [11–12] (Figure 2E).

To measure NT, we confirmed the line connecting the upper end of the sternum and the center of the T1 upper endplate. One edge of the smartphone was aligned to this line, and the absolute value of this angle displayed on the smartphone screen was recorded as NT (or equal to 90° minus the absolute value of this angle displayed on the smartphone screen if NT > 45°) [11–12] (Figure 2F).

Two observers (one of them was a spine specialist and the other was a resident of general orthopedics) reviewed the films independently and blinded. The two observers measured the four representative angles using a smartphone’s integrated inclinometer to evaluate the interobserver reliability. To assess intraobserver reliability, observer A measured the parameters again using the same smartphone method. To assess the validity of the smartphone measurement, observer A measured the parameters using PACS and compared them to the previous outcomes measured by a smartphone [12,16]. The order of the films was disorganized to reduce bias. A stopwatch was used to record the time of every measurement. To simulate the real operation scenario, the measurement time of the PACS is defined as the time interval between the input of the X-ray number in the system and the recording of the angle, and the measurement time of the smartphone is defined as the time interval between the inclinometer turning on and recording of the angle [12,16]. Data were recorded in Excel 2016.

### 2.3. Statistic analysis

SPSS software (version 21.0) was used for the data analysis. Reliability (interobserver and intraobserver) was evaluated using intraclass correlation coefficients (ICCs) [12,16]. Observer A measured the parameter using PACS and compared the result to the mean result of two measurements of the smartphone to assess the validity of the novel method [12,16]. Observer A compared the results of two measurements using this integrated inclinometer method to evaluate intraobserver reliability [12,16]. Observer B made the measurement only once and compared the outcome to the average outcome of two measurements of the integrated inclinometer method by observer A to assess the interobserver reliability [12,16]. Over 0.75 is identified as good ICC, over 0.85 is identified as very good ICC, an excellent ICC is identified as over 0.9 [16]. To visualize the reliability (interobserver and ntraobserver) and validity of this integrated inclinometer method, we used MedCalc software to draw the Bland-Altman plot showing the 95% confidence interval (CI) and mean difference [12,16]. The time taken for measurement by the PACS method was recorded and compared to the average time of two measurements by the integrated inclinometer method from observer A, to assess the time difference between the two methods conducted by independent-samples T test [12,16].

## 3. Results

### 3.1. Basic outcomes

A total of 120 lateral plain films (56 males and 64 females) were reviewed from patients with cervical spondylosis (myelopathy or radiculopathy). The mean age was 57.0 ± 10.9 years old. The average results of C0-2 cobb angle, C2-7 cobb angle, T1S and NT were 21.72 ± 7.68°, 15.25 ± 9.35°, 23.99 ± 7.49° and 52.29 ± 6.57° respectively measured by PACS. The measurement by smartphone’s integrated inclinometer (46.31 ±3.99 s) was significantly quicker than that by PACS (69.48 ± 3.25 s) according to independent-samples T test (*p* < 0.001). The mean difference is 23.17s and 95% CI ranges from 22.24 to 24.09, indicating that our novel smartphone method is faster compared to PACS method.

### 3.2. Validity

**Figure 3.**
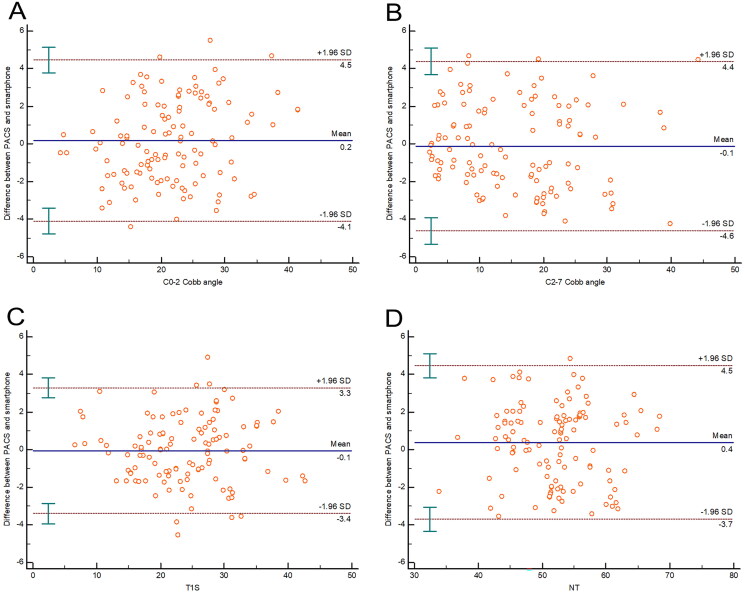
The Bland-Altman plot displayed the validity for the represented parameters. The middle full line represented the mean difference, and the other dashed lines represented the 95% CI.

For the representative parameters (C0-2 Cobb angle, C2-7 cobb angle, T1S, and NT), the ICC were 0.957 (0.939-0.970), 0.971 (0.958-0.979), 0.974 (0.963–0.982) and 0.949 (0.927–0.964) for validity respectively. For the validity of the representative parameters mentioned above, the Bland-Altman plot displayed a mean difference of 0.2, 0.1, 0.1, and 0.4°with a 95% CI of 4.3, 4.5, 3.4, and 4.1°, respectively (Figure 3).

### 3.3. Intraobserver reliability

**Figure 4.**
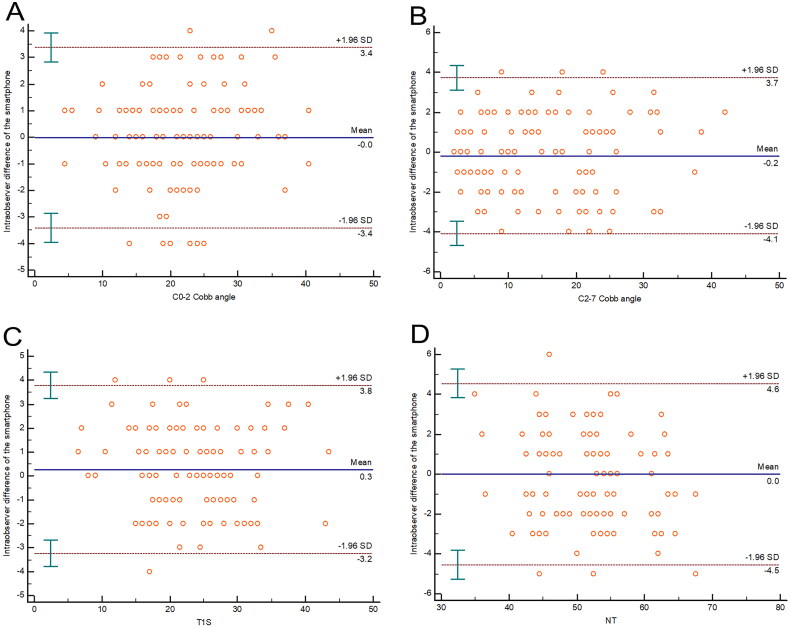
The Bland-Altman plot displayed the intra-observer reliability of the smartphone measurement for the represented parameters. The middle full line represented the mean difference, and the other dashed lines represented the 95% CI.

For the aforementioned representative parameters, the ICC values were 0.972 (0.960–0.980), 0.979 (0.969–0.985), 0.972 (0.959–0.980), 0.942 (0.917–0.959) for intraobserver reliability respectively. For intraobserver reliability of the representative parameters mentioned above, the Bland-Altman plot displayed a mean difference of 0.0, 0.2, 0.3, and 0.0°with a 95% CI of 3.4, 3.9, 3.5, and 4.6°, respectively (Figure 4).

### 3.4. Interobserver reliability

**Figure 5.**
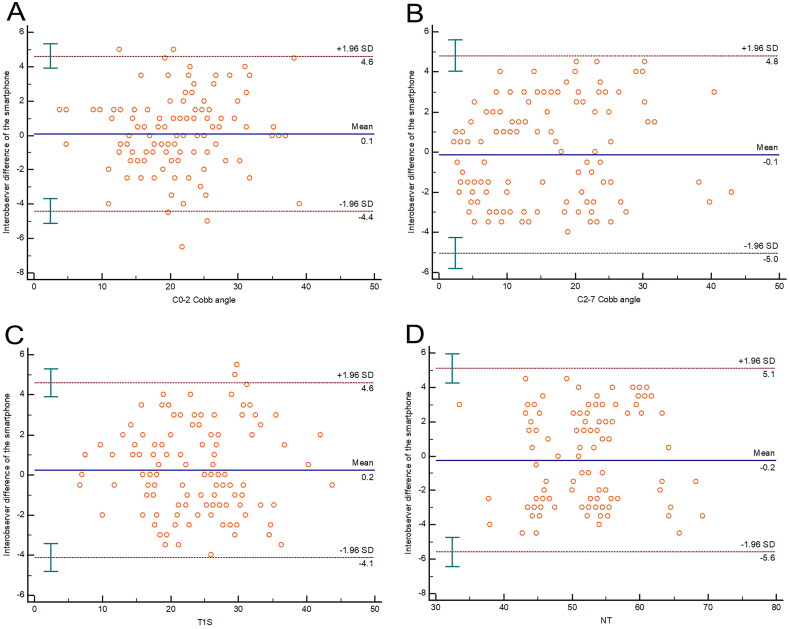
The Bland-Altman plot displayed the inter-observer reliability of the smartphone measurement for the represented parameters. The middle full line represented the mean difference, and the other dashed lines represented the 95% CI.

For the representative parameters mentioned above, the ICC values were 0.947 (0.926–0.963), 0.964 (0.949–0.975), 0.956 (0.938–0.969), 0.916 (0.881–0.940) for interobserver reliability respectively. For interobserver reliability of the representative parameters mentioned above, the Bland-Altman plot displayed a mean difference of 0.1, 0.1, 0.2, and 0.2°with a 95% CI of 4.5, 4.9, 4.4, and 5.4°, respectively (Figure 5).

## 4. Discussion

An angulometer and marker pen are required in traditional manual methods, which are not always carried around by doctors. The shortcomings of this traditional process include the time required, film staining, and fallibility [17]. With advances in computer technology, PACS has been used as the gold standard for measuring orthopedic parameter angles. However, PACS in different hospitals are always incompatible with each other. Patients admitted to one hospital always carry radiographic films from other hospitals, which cannot be operated on and measured in the hospital’s PACS [9–10]. With the development of smartphones, some apps have been invented to measure orthopedic angles to improve the portability and social character of PACS [18–19]. The angles of films from other hospitals could also be easily measured by these apps. However, these apps still have weaknesses, including unstable and possible flashback, download fees, lack of maintenance and upgrading, and incompatibility with various mobile phone systems [11].

Therefore, in clinical practice, we used a new simple method for measuring cervical angles based on the smartphone’s integrated inclinometer. The purpose of the inclinometer is to measure the tilt angle, which is rooted in almost every smartphone as an intrinsic function. In our previous study, this method had excellent validity and reliability for measuring thoracolumbar kyphotic angles [11]. However, the reliability and validity of this smartphone method for measuring the sagittal angles of the cervical spine are still unknown. Therefore, we conducted a comparative analysis. The results of the present study revealed that the validity and intra- and inter-observer reliability of the integrated inclinometer are excellent for the measurement of all representative parameters. Carman et al. found that when reviewing lateral radiographs of the spine for Cobb angle, 11° was supposed to represent a 95% CI for measurement difference to represent a true change [20]. Another study showed that 5.6° was the minimum detectable change in T1S [21]. Almost all the results in this study were within acceptable limits, according to the visualization from the Bland-Altman plot. It is noteworthy that the reliability (inter- and intra-observer) and validity of NT were all lower than those of the other representative para­meters. This may be because the two points (the sternum’s upper end and the center of the T1 upper endplate) that make up the connecting lines of NT were not very clear even on the selected X-ray in the present study. In addition, the measurement time of this smartphone method using the built-in inclinometer was much shorter than that of the PACS. The use of this intrinsic inclinometer also has other advantages such as being free, no need to download, no need to update, this intrinsic function is smoother and more stable than downloaded apps, has more socialization properties, and is easy to consult with other doctors [12].

Because of the unclear X-ray film, it is difficult to identify the endplate of the vertebra and other anatomic landmarks, which leads to potential measurement errors when using this smartphone method. This bias also exists in the PACS and traditional measurement processes. The innovation of this method does not change the principles and spirit of the measurements, and the inherent measurement error remains [12,22]. Additionally, some key points must be emphasized during the measurement process. Owing to the principle of the inclinometer, the measured radiographic X-ray films should be located plumbs to avoid errors. Because of the similar spirit and steps, the measurement of CT or MRI using this smartphone method may also be reliable, and further studies should be conducted to confirm this hypothesis. Even though all the films in this study were from patients with cervical spondylosis, this method should be applied to measure angles from the normal population because of the same measurement steps. In addition, sufficient exercise and practice are needed for accurate and rapid measurements using this smartphone method.

## 5. Conclusion

The validity and intra- and inter-observer reliabilities of this integrated inclinometer method were excellent. It also has advantages in terms of saving time. In summary, this novel smartphone measurement method based on an integrated inclinometer is accurate and reliable for rapidly and conveniently measuring cervical sagittal parameters.

## Data Availability

The datasets used and/or analysed during the current study available from the corresponding author on reasonable request.

## References

[1] Tang R, Ye IB, Cheung ZB, et al. Age-related changes in cervical sagittal alignment: a radiographic analysis. Spine. 2019;44(19):1–8. doi: 10.1097/BRS.0000000000003082. 31261278

[2] Xing R, Liu W, Li X, et al. Characteristics of cervical sagittal parameters in healthy cervical spine adults and patients with cervical disc degeneration. BMC Musculoskelet Disord. 2018;19(1):37. doi: 10.1186/s12891-018-1951-8. 29390994 PMC5795794

[3] Rostami M, Moghadam N, Obeid I, et al. The impact of Single-Level anterior cervical discectomy and fusion on cervical sagittal parameters and its correlation with pain and functional outcome of patients with neck pain. Int J Spine Surg. 2021;15(5):899–905. doi: 10.14444/8115. 34625454 PMC8651199

[4] Woodroffe RW, Helland L, Hollatz C, et al. Impact of the inclusion of C2 in posterior cervical fusions for cervical myelopathy on sagittal cervical alignment. Clin Spine Surg. 2020;33(4):E141–E146. doi: 10.1097/BSD.0000000000000931. 31913172

[5] Wu J, Guo R, Yang C, et al. The difference of sagittal correction of adult subaxial cervical spine surgery according to age: a retrospective study. Orthop Surg. 2022;14(8):1790–1798. doi: 10.1111/os.13385. 35819084 PMC9363747

[6] Wang WX, Zhao YB, Lu XD, et al. Influence of extending expansive open-door laminoplasty to C1 and C2 on cervical sagittal parameters. BMC Musculoskelet Disord. 2020;21(1):75. doi: 10.1186/s12891-020-3083-1. 32024507 PMC7003532

[7] Li J, Zhang D, Shen Y. Impact of cervical sagittal parameters on axial neck pain in patients with cervical kyphosis. J Orthop Surg Res. 2020;15(1):434. doi: 10.1186/s13018-020-01909-x. 32962694 PMC7509936

[8] Lee SH, Hyun SJ, Jain A. Cervical sagittal alignment: literature review and future directions. Neurospine. 2020;17(3):478–496. doi: 10.14245/ns.2040392.196. 33022153 PMC7538362

[9] Huang T, Wang L, Lu C, et al. A novel rapid measurement of hallux valgus parameters using the built-in photo edit function of smartphones. BMC Musculoskelet Disord. 2021;22(1):716. doi: 10.1186/s12891-021-04604-y. 34419028 PMC8380395

[10] Wang L, Zhang C, Liang H, et al. Reliability of different smartphones measuring the hallux valgus parameters in a new rapid method: a follow-up study. BMC Musculoskelet Disord. 2022;23(1):315. doi: 10.1186/s12891-022-05217-9. 35366850 PMC8976351

[11] Huang T, Zhao Z, Wang L, et al. Rapid measurement of thoracolumbar kyphosis with the integrated inclinometer of a smartphone: a validity and reliability study. Sci Rep. 2022;12(1):8745. doi: 10.1038/s41598-022-12690-8. 35610284 PMC9130239

[12] Zhang J, Zhang C, Zhong W, et al. Validity and reliability of a novel iPhone method to rapidly measure cervical sagittal parameters. Sci Rep. 2022;12(1):19579. doi: 10.1038/s41598-022-21660-z. 36380107 PMC9666521

[13] Kim TH, Lee SY, Kim YC, et al. T1 slope as a predictor of kyphotic alignment change after laminoplasty in patients with cervical myelopathy. Spine. 2013;38(16):E992–997. doi: 10.1097/BRS.0b013e3182972e1b. 23609205

[14] Morimoto Y, Shigematsu H, Iwata E, et al. Evaluating cervical sagittal alignment in cervical myelopathy: are sitting cervical radiographs and standing whole-Spine radiographs equally useful? Global Spine J. 2019;9(6):591–597. doi: 10.1177/2192568218811841. 31448191 PMC6693062

[15] Hu L, Lv Y, Lin Y. Correlations and age-related changes of cervical sagittal parameters in adults without symptoms of cervical spinal disease. Spine . 2020;45(23):E1542–E1548. doi: 10.1097/BRS.0000000000003680. 32890305

[16] Balg F, Juteau M, Theoret C, et al. Validity and reliability of the smartphone to measure rib hump in scoliosis. J Pediatr Orthop. 2014;34(8):774–779. doi: 10.1097/BPO.0000000000000195. 24787301

[17] Wang J, Chen T, Rui X, et al. Rapid measurement of lumbosacral spine-pelvic sagittal balance parameters using electronic device. J King Saud Univ Sci. 2020;32(8):3217–3222. doi: 10.1016/j.jksus.2020.03.036.

[18] Jacquot F, Charpentier A, Khelifi S, et al. Measuring the cobb angle with the iPhone in kyphoses: a reliability study. Int Orthop. 2012;36(8):1655–1660. doi: 10.1007/s00264-012-1579-5. 22653103 PMC3535038

[19] Lee JB, Kim IS, Lee JJ, et al. Validity of a smartphone application (sagittalmeter pro) for the measurement of sagittal balance parameters. World Neurosurg. 2019;126:e8–e15. doi: 10.1016/j.wneu.2018.11.242. 30557655

[20] Carman DL, Browne RH, Birch JG. Measurement of scoliosis and kyphosis radiographs. Intraobserver and interobserver variation. J Bone Joint Surg Am. 1990;72(3):328–333. doi: 10.2106/00004623-199072030-00003. 2312528

[21] Marques C, Granstrom E, MacDowall A, et al. Accuracy and reliability of x-ray measurements in the cervical spine. Asian Spine J. 2020;14(2):169–176. doi: 10.31616/asj.2019.0069. 31668048 PMC7113471

[22] Cobb JR. The problem of the primary curve. J Bone Joint Surg Am. 1960;42-A:1413–1425. 14448728

